# Rosiglitazone synergizes anticancer activity of cisplatin and reduces its nephrotoxicity in 7, 12-dimethyl benz{a}anthracene (DMBA) induced breast cancer rats

**DOI:** 10.1186/1471-2407-9-107

**Published:** 2009-04-08

**Authors:** Kulbhushan Tikoo, Parveen Kumar, Jeena Gupta

**Affiliations:** 1 Laboratory of Chromatin Biology, Department of Pharmacology and Toxicology, National Institute of Pharmaceutical Education andResearch (NIPER), Sector 67, S.A.S. Nagar, Punjab-160 062, India

## Abstract

**Background:**

Antineoplastic drug cisplatin remains the drug of choice for various solid tumours including breast cancer. But dose dependent nephrotoxicity is the major drawback in majority of platinum based chemotherapy regimens. Recent reports have shown that inflammatory pathways are the main offender for cisplatin induced nephrotoxicity. The present study was undertaken to assess the effect of rosiglitazone, a PPARγ agonist and an anti-inflammatory agent, on cisplatin induced nephrotoxicity, and its anticancer activity in DMBA induced breast cancer rats.

**Methods:**

Mammary tumours were induced in female Sprague-Dawley rats by feeding orally with dimethylbenz [a]anthracene (DMBA) (60 mg/kg). Cisplatin induced nephropathy was assessed by measurements of blood urea nitrogen, albumin and creatinine levels. Posttranslational modifications of histone H3, mitogen-activated protein (MAP) kinase p38 expression and PPAR-γ expression were examined by western blotting.

**Results:**

Our data shows involvement of TNF-α in preventing cisplatin induced nephrotoxicity by rosiglitazone. Rosiglitazone pre-treatment to cisplatin increases the expression of p38, PPAR-γ in mammary tumours and shows maximum tumour reduction. Furthermore, cisplatin induced changes in histone acetylation, phosphorylation and methylation of histone H3 in mammary tumours was ameliorated by pre-treatment of rosiglitazone. Suggesting, PPAR-γ directly or indirectly alters aberrant gene expression in mammary tumours by changing histone modifications.

**Conclusion:**

To best of our knowledge this is the first report which shows that pre-treatment of rosiglitazone synergizes the anticancer activity of cisplatin and minimizes cisplatin induced nephrotoxicity in DMBA induced breast cancer.

## Background

Breast cancer is a complex disease that results from a multi-stage process involving the deregulation of a number of different signalling cascades. About 212,930 new cases of breast cancer were diagnosed every year, of which 40,840 were related to deaths in the United States alone [1]. This continuing magnitude of the breast cancer problem with respect to incidence, morbidity and mortality requires further studies involving novel approach to prevent this disease [2]. Cisplatin, cis- [PtCl2(NHs)2], is a widely used anticancer drug, proved to be beneficial in the treatment of wide variety of solid tumours (head and neck, lung, bladder, colorectal and breast cancer) in various combination chemotherapy regimens [3]. Higher doses of cisplatin are considered to be more efficacious for cancer chemotherapy; however these therapies manifest toxicities such as nephrotoxicity, despite its effectiveness which limits its use [4,5]. It was observed that after a single injection of cisplatin, around 28 to 36% of patients develop dose-dependent nephrotoxicity [6]. However despite its toxicity, cisplatin remains to be one of the most commonly used chemotherapy drugs due to its therapeutic efficacy [7]. Recent reports have shown that cisplatin induced nephrotoxicity is characterized by activation of pro-inflammatory cytokines and chemokines. TNF-α appears to play a central role in the cisplatin induced renal injury by activation of a large network of chemokines and cytokines in the kidney following cisplatin injection. Blockade of either TNF-α production or its activity prevents the activation of cytokine network and provides protection against cisplatin-induced renal dysfunction and structural damage [8,9].

Peroxisome proliferator-activated receptor-γ(PPARγ) is a member of the nuclear receptor superfamily of ligand-activated transcription factors [10]. PPARγ forms a heterodimeric complex with the retinoid × receptor and then binds to the PPAR response element [11]. This interaction can be responsible for the regulation of cellular events ranging from glucose and lipid homeostasis to cell differentiation and apoptosis [12]. Ligands for PPARγ include natural compounds such as fatty acids and their derivatives and synthetic agents such as the antidiabetic drugs rosiglitazone (Avandia) and pioglitazone (Actos) [13]. Although central role of PPAR-γ has been demonstrated in the differentiation of adipose cells, PPARγ has also been shown to regulate the growth, differentiation, and gene expression in number of different cancer cells [14,15]. Rosiglitazone has been shown to have anti-neoplastic activity in *in-vitro *and *in-vivo *breast cancer models [16]. In addition, recently, several studies have demonstrated that PPARγ agonist's exhibit an anti-inflammatory effect *in-vitro *and *in-vivo *[17,18].

The eukaryotic genome is maintained as a nucleoprotein complex called chromatin, which consists, mainly of positively charged proteins called histones [19]. Post-translational modification of histone proteins as well as non-histone proteins including nuclear receptors integrates signalling pathways mediating diverse biological processes. The influence exerted by the post-translational modifications of histones over the regulation of gene expression has been extensively studied in the past few years [20]. Post-translational modification of histones provides a key mechanism of transcriptional regulation. Acetylation, phosphorylation and methylation of histone H3, modulate the activity of many genes by modifying both core histones and non-histone protein [21]. Alterations in modifications of histones have been linked to deregulation of many genes which have important roles in cancer development and progression [22]. Moreover, it has been reported that HER2-overexpressing breast cancer cells contained significantly higher levels of acetylated and phosphorylated histone H3 [23]. So far there are no reports regarding the effect of rosiglitazone pretreatment on post-translational histone modifications in DMBA induced breast cancer in female SD rats.

Based upon above facts we hypothesize that pre-treatment of rosiglitazone along with cisplatin decreases its nephrotoxicity and may synergize or potentiate its anticancer activity. Understanding the mechanism of rosiglitazone and cisplatin treatment will help in initiating novel approach for cancer therapeutics.

## Methods

### Materials

Cisplatin and Rosiglitazone were kindly gifted by Dabur India Pvt. Ltd and Nicholas Piramal Research Centre, Mumbai, India, respectively. DMBA was obtained from Sigma (St. Louis, MO, USA). Blood urea nitrogen (BUN), creatinine and albumin kits were purchased from Accurex (Mumbai, India).

### Animals

The guidelines of committee for the purpose of control and supervision of experiments on animals (CPCSEA), Government of India were followed and prior permission was sought from the institutional animal ethics committee (IAEC) for conducting the study. The female Sprague-Dawley rats (160–180 g) were procured from the central animal facility of the institute. They were maintained under standard environmental conditions and provided with feed and water *ad libitum*. All the animals were fed on normal pellet diet one week prior to the experimentation. Considering the animal ethical issues, all animals were kept under best hygienic conditions and the tumour bearing animals were inspected daily for any signs of pain, discomfort of distress.

### Tumour induction

Female Sprague Dawley rats at the age of 8 weeks weighing 160–180 g were gavaged with 60 mg dimethylbenz [a]anthracene (DMBA)/kg body weight, a dose sufficient to cause 100% tumour incidence in the control group over the course of the study as described by Whitsett T *et al *[24]. The DMBA was dissolved in olive oil at a stock solution of 30 mg/ml. Animals were sacrificed when the tumour diameter reached three inch, animals became moribund or after the completion of experiment.

### Experimental design

Female SD rats were divided initially into two different groups namely, normal control (Group I) received olive oil and DMBA treated group received DMBA (60 mg/kg). After three months mammary carcinoma was confirmed by histological examination and breast palpation. Twelve weeks later, DMBA treated rats were grouped into four different groups on the basis of their tumour volume. DMBA treated rats received normal saline (Group II). Mammary carcinoma induced rats treated with rosiglitazone (8 mg/kg) suspended in 0.5% CMC through oral gavage (Group III). Mammary carcinoma induced rats treated with cisplatin (7.5 mg/kg) dissolved in normal saline by intraperitoneal route (Group IV). Mammary carcinoma induced rats first pre-treated with rosiglitazone (8 mg/kg) suspended in 0.5% CMC for five days through oral gavage and then on fifth day single dose of cisplatin (7.5 mg/kg) dissolved in normal saline (0.9% w/v) by intra-peritoneal route (Group V). These animals were maintained on standard diet and water for 3 months prior to drug ingestion.

### Estimation of plasma glucose, albumin, blood urea nitrogen and creatinine

Blood samples were collected from rat tail vein under light ether anesthesia in heparinized centrifuge tubes and immediately centrifuged at 2300 g for the separation of plasma. Plasma was stored at -80° until assayed. The plasma was used for the estimation of albumin, blood urea nitrogen (BUN) and creatinine as described previously [25].

### Measurement of TNF-α level

The level of TNF-α in serum was determined by using enzyme-linked immunosorbent assay (ELISA) kits (Endogen, Woburn, MA, USA), according to the manufacturer's instructions. In all the cases, a standard curve was constructed from standards provided by the manufacturer.

### Measurement of tumour volume

The measurements were done for visible tumours; two diameters that is shortest and longest diameter of the tumours were measured. The volume of the tumour was calculated as: Π/6.(a)^2^(b), where a is the smallest and b is the longest length of the tumour [26].

### Histopathology of Mammary and kidney

Histopathology of kidneys and mammary tumours were done as described previously [27,28]. Briefly rats were anesthetized under light ether anesthesia, after surgery circulating blood was removed by cardiac perfusion with 0.1 M PBS (pH 7.4; 20–50 ml). After clearance of circulating blood, 4% paraformaldehyde in 0.1 M phosphate buffer (pH 7.4) was perfused for another 5 min (100–200 ml of fixative) to fix the tissues. Kidneys and mammary tumours were removed from the animal, decapsulated, sliced transversely, and paraffin embedded for light microscopic evaluation. Histopathological changes in these tissues were assessed in at least 25 randomly selected tissue sections from each group studied. Sections were stained with Meyer's hematoxylin and eosin to examine cell structure. Slides were observed under Olympus microscope (Model no. BX51). For histological quantification 100 renal tubules were randomly examined from each animal and scored as 0, 1, 2, and 3 (No damage, Mild, Moderate, and extensive damage) depending on the extent of damage observed. The no. of tubules under each score was multiplied with the respective scores and the sum obtained to get the final renal tubules damage score. For histological quantification of mammary tumours the histological grades were assessed as proposed by Elston and Ellis[29]. Briefly histological grade was obtained by summing the scores of three parameters: tubular formation, nuclear pleomorphism and mitotic counts and then classified as follows: 3–5 points: grade I, well differentiated; 6–7 points: grade II, moderately differentiated; 8–9 points: grade III, poorly differentiated.

### Protein isolation and western blotting

Nuclei, histone isolation and western blotting were performed in kidney tissues as described previously [25,27,28]. Immunoblot analysis was performed by using Anti phospho-Histone H3 (ser-10) (rabbit 1:2000, Santa Cruz, CA), Anti acetyl-Histone H3 (lysine 14) (rabbit 1:2000, Santa Cruz, CA), Anti Histone H3 (rabbit 1:5000, Upstate, Lake Placid, NY), Anti PPARγ (rabbit 1:500, Santa Cruz, CA), Anti p38 (rabbit 1:500, Santa Cruz, CA), Anti-actin (rabbit 1:2500, Sigma, St. Louis, MO) and HRP-conjugated secondary antibodies (anti-rabbit) from Santa Cruz. Proteins were detected with the enhanced chemiluminescence system and ECL Hyperfilm (Amersham Pharmacia Biotech, UK Ltd, Little Chalfont, Buckinghamshire, UK).

### Statistical analysis

Experimental values are expressed as mean ± SEM. Comparison of mean values between various groups was performed by one way-analysis of variance (one way-ANOVA) followed by multiple comparisons by Turkey test. P-value < 0.05 is considered to be significant.

## Results

### Effect of rosiglitazone on cisplatin induced change in body weight, BUN, plasma creatinine and plasma albumin levels

Treatment of cisplatin show significant loss in body weight when compared with breast cancer control rats. However, there was no significant change in body weight of rosiglitazone treated rats. Pretreatment of rosiglitazone (8 mg/kg) for five days in cisplatin treated rats showed significant protection in body weight loss as compared to cisplatin (7.5 mg/kg) treated rats in breast cancer animals. In comparison with cancer control rats, nephropathy markers like BUN and creatinine levels, show significant increase by cisplatin treatment and a decrease in albumin level. Pretreatment of rosiglitazone (8 mg/kg) for five days before cisplatin (7.5 mg/kg) treatment showed significant decrease in BUN and creatinine levels and increased albumin levels as compared to cisplatin (7.5 mg/kg) treated rats in breast cancer animals. There was no significant change in BUN, creatinine and albumin levels in rosiglitazone (8 mg/kg) treated rats (Table 1). These observations demonstrate that rosiglitazone pre-treatment is effective in reducing cisplatin induced nephrotoxicity.

**Table 1:** Effect of rosiglitazone pre-treatment on cisplatin-induced nephrotoxicity in chemically induced breast cancer model

	Change in Body weight (g)	BUN (mg/dl)	Creatinine (mg/kg)	Albumin (g/dl)
**Normal control**	11.00 ± 3.37	33 ± 3.78	1.02 ± 0.08	3.59 ± 0.03

**Breast cancer control**	3.00 ± 3.12	36 ± 3.53	1.07 ± 0.12	3.73 ± 0.12

**Rosi (8 mg/kg)**	8.00 ± 3.49	28 ± 2.85	0.93 ± 0.05	3.58 ± 0.09

**Cis (7.5 mg/kg)**	-35.00 ± 2.52***^a^	318 ± 14.20***^a^	5.52 ± 0.35***^a^	2.94 ± 0.14***^a^

**PT Rosi (8 mg/kg) + Cis (7.5 mg/kg)**	-6.00 ± 2.33***^b^	167 ± 14.41***^b^	1.96 ± 0.23***^b^	3.73 ± 0.20***^b^

All the values are expressed as mean ± SEM (n = 8). ***P < 0.001, a Vs Cancer Control & b Vs Cisplatin. Where, Rosi is rosiglitazone, Cis is cisplatin and PT is pretreatment

### Rosiglitazone prevents cisplatin induced increase in TNF-α level in breast cancer rats

**Figure 1:**
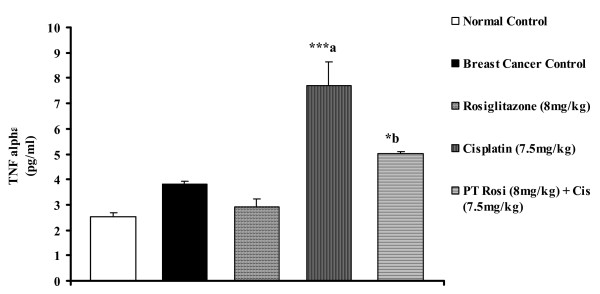
**Effect of rosiglitazone pretreatment on level of TNFα in serum following cisplatin administration**. All values are expressed as mean ± SEM (n = 8). ***P < 0.001, *P < 0.05. a Vs Cancer Control & b Vs Cisplatin.

Previous reports have suggested that TNF-α is involved in cisplatin induced nephrotoxicity and that a blockade of TNF-α action ameliorates cisplatin induced nephrotoxicity [8]. We measured the level of TNF-α in serum at 5^th ^day after the administration of rosiglitazone, cisplatin or cisplatin with rosiglitazone pretreatment. Cisplatin injection increased TNF-α in serum on 5^th ^day as compared to breast cancer control group. However, pretreatment with rosiglitazone significantly decreased serum TNF-α levels in the cisplatin-treated group (Fig. 1). Suggesting that nephroprotection observed by rosiglitazone involves TNF-α inhibition.

### Pretreatment of rosiglitazone potentiates antitumour activity of cisplatin in DMBA induced breast cancer rats

#### Tumour volume

Table 2 represents the tumour volume in rats after treatment with cisplatin, rosiglitazone, and combination of both. There was a considerable increase in tumour volume of breast cancer control animals when compared with the treatment group's like cisplatin, rosiglitazone and pre-treatment group. Cisplatin and rosiglitazone treated rats showed a significant reduction in their tumour volume when compared with untreated rats. Reduction in tumour volume of cisplatin treated animals was more as compared with rosiglitazone treated animals. However, rosiglitazone pretreatment along with cisplatin show maximum reduction in tumour volume as compared to cisplatin alone treated rats. These finding suggest us that combination of rosiglitazone and cisplatin is more effective than individual therapies.

**Table 2:** Effect of pre-treatment of rosiglitazone with cisplatin on tumour volume (cm) in chemically induced breast cancer model

	0 Day	3^rd ^Day	6^th ^Day	9^th ^Day
**Normal control**	0 ± 0	0 ± 0	0 ± 0	0 ± 0

**Breast cancer control**	2.3 ± 1.04	4.33 ± 0.99	5.85 ± 1.16	6.5 ± 1.46

**Rosi (8 mg/kg)**	2.26 ± 0.48	1.94 ± 0.61	1.89 ± 0.73**^a^	1.85 ± 0.19**^a^

**Cis (7.5 mg/kg)**	2.23 ± 0.57	1.85 ± 0.44	1.62 ± 0.41**^a^	1.53 ± 0.35***^a^

**PT Rosi (8 mg/kg) + Cis (7.5 mg/kg)**	2.06 ± 0.84	1.63 ± 0.56*^a^	1.25 ± 0.46**^a^	0.98 ± 0.36***^a^

All values are expressed as mean ± SEM (n = 8). ***P < 0.001, **<0.01, *P < 0.05. a Vs Cancer Control. Where Rosi is rosiglitazone, Cis is cisplatin and PT is pretreatment

#### Percentage tumour inhibition

Percentage tumor inhibition was calculated as compared to cancer control in respective treatment groups (Table 3). There was a considerable tumour progression in untreated rats when compared with treated rats. Tumour did not disappear totally by single dose of drug treatment, but a significant regression was observed when compared with untreated rats. In comparison to breast cancer control rats, Cisplatin treated rats showed 82.95% reduction on the 3^rd ^day, 72.64% reduction on 6^th ^day and 68.60% reduction on 9^th ^day of the tumour size. Rosiglitazone treated rats showed 85.84, 83.62 and 81.85% reduction on 3^rd ^day, 6^th ^and 9^th ^days, respectively. Moreover combination therapy treated rats showed a 79.12, 60.67 and 54.85% reduction of tumour size on 3^rd^, 6^th ^and 9^th ^days, respectively, indicating combination therapy was most effective when compared to rosiglitazone and cisplatin alone treated rats.

**Table 3:** Effect of pre-treatment of rosiglitazone in cisplatin treated rats on % tumour inhibition in chemically induced breast cancer model

	0 Day	3^rd ^Day	6^th ^Day	9^th ^Day
**Normal control**	0 ± 0	0 ± 0	0 ± 0	0 ± 0

**Breast cancer control**	100 ± 45.22	188.26 ± 22.86	254.34 ± 19.83	282.60 ± 22.46

**Rosi (8 mg/kg)**	100 ± 21.24	85.84 ± 31.44	83.62 ± 38.62**^a^	81.85 ± 10.27**^a^

**Cis (7.5 mg/kg)**	100 ± 25.56	82.95 ± 23.78	72.64 ± 25.31**^a^	68.60 ± 22.88***^a^

**PT Rosi (8 mg/kg) + Cis (7.5 mg/kg)**	100 ± 40.78	79.12 ± 34.36*^a^	60.67 ± 36.80**^a^	54.85 ± 31.86***^a^

Percentage tumor inhibition is assessed by calculating the percentage of reduction in tumor volume in the respective treated groups as compared to breast cancer control All values are expressed as mean ± SEM (n = 8). ***P < 0.001, **<0.01, *P < 0.05. a Vs Cancer Control. Where Rosi is rosiglitazone, Cis is cisplatin and PT is pretreatment.

### Changes in renal histology by cisplatin and rosiglitazone treatment in breast cancer rats

**Figure 2:**
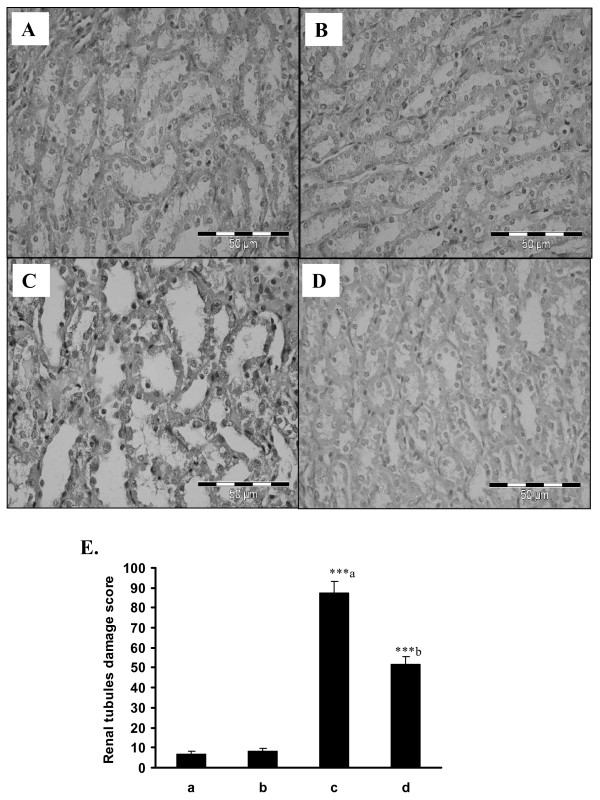
**Histopathological changes in kidney after pre-treatment with rosiglitazone**. Transverse section of normal control rat kidney (A), rosiglitazone treated kidney (B), cisplatin treated (C) and pre-treatment of rosiglitazone (D). Sections were stained with Mayer's hematoxylin counterstained with eosin and observed under magnification of 40× as described in Section 2. (E) Quantitative analysis of histopathological studies in kidney where; control rat kidney (a), rosiglitazone treated kidney (b), cisplatin treated (c) and pre-treatment of rosiglitazone (d).

Cancer control rats showed intact renal tubules and glomeruli, in addition, uniform tubules with single layer of epithelium lining was observed in renal cortex of control rats (Fig. 2A). Cisplatin treated rats showed increased tubular space, vacuolation and desquamation of epithelial cells in renal tubules (Fig 2C). However, pre-treatment with rosiglitazone dramatically improved the cisplatin nephrotoxicity showing minimum tubular damage in this group (Fig. 2D). Rosiglitazone treatment alone (Fig. 2B) had no effect on renal histology. Supporting our data of biochemical protection observed in cisplatin induced nephrotoxicity.

### Effect of rosiglitazone pre-treatment on mammary tumour histology in DMBA induced breast cancer

**Figure 3:**
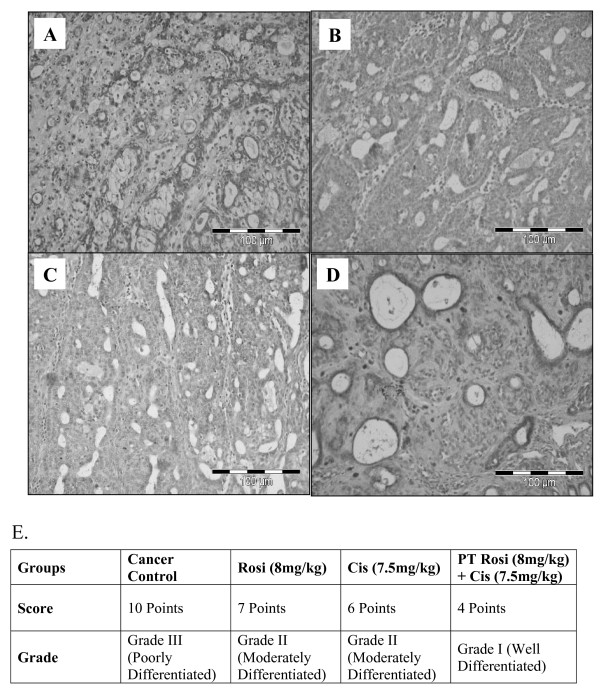
**Effect of rosiglitazone pretreatment on mammary tumour histopathology**. (A) Breast cancer control group, (B) Mammary tumour after treatment with Rosiglitazone: pronounced cell pleomorphism and a low grade of differentiation are demonstrated by multinucleated giant cells, (C) Cisplatin treated mammary tumour. Note the decreased cell density and higher level of fibrosis as sign of a therapeutic effect, (D) Rosiglitazone pre-treated mammary tumour for five days. Note the glandular structure as indicator for a functional differentiation, (E) Quantitative analysis of histopathological studies in mammary tumour where; Cancer control (a), rosiglitazone treated mammary tumour (b), cisplatin treated mammary tumour (c) and rosiglitazone pretreated mammary tumour (d).

Breast cancer control group displays normal neoplasm histology (Fig. 3A). Rosiglitazone treated group showed, a low grade of differentiation which is demonstrated by giant multinucleated cells (Fig. 3B). Decreased cell density and higher level of fibrosis was observed in cisplatin treated animals (Fig. 3C). However, Rosiglitazone pre-treatment for five days in cisplatin treated animals showed appearance of glandular structures, an indication for functional differentiation (Fig. 3D). Suggesting combination of cisplatin and rosiglitazone prevents tumour progression significantly.

### Rosiglitazone changes p38 expression in mammary tumour

**Figure 4:**
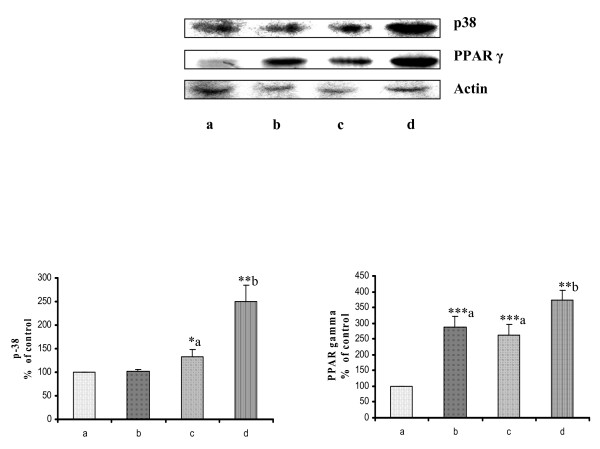
**Western blot of PPARγ and MAP kinase p38 in mammary tumours**. Western blot of PPARγ and MAP kinase p38 levels in mammary tumours after treatment with Rosiglitazone and Cisplatin in DMBA induced breast cancer rats. Where, lane a: Cancer Control, lane b: Rosiglitazone, lane c: Cisplatin and lane d: Rosiglitazone pre-treatment. Results were normalized with respect to Actin. Similar results were obtained in three independent set of experiments. All the values were represented as Mean ± S.E.M. (n = 3), **P < 0.01; * P < 0.05; a Vs Cancer Control and b Vs Cisplatin.

It has been shown that p38 participates in the regulation of apoptotic cell death through transcriptional upregulation of proapoptotic gene expression, such as Fas ligand [30,31]. p38alpha can suppress cell proliferation by antagonizing the JNK/c-Jun pathway, which is an important regulator of proliferation and apoptosis [32]. In addition, recent reports show that p38 phosphorylation is down regulated and Akt phosphorylation is up regulated in multiple human tumour tissues, and this correlates with tumour stage in human breast cancer [33]. Cancer control animals showed very low level of p38 (Fig. 4 lane a). Rosiglitazone and cisplatin (Fig. 4 lane b and c) treated animals showed increased p38 in mammary tumour when compared with cancer control. However, pretreatment of rosiglitazone, showed further increase in level of p38 expression, suggesting further increase in apoptotic cell death in breast cancer cells. The increase in the p38 expression supports our earlier data of maximum tumour reduction in pre-treatment group.

### Rosiglitazone pretreatment in breast cancer increases PPARγ expression

It has been previously reported that PPARγ is detectable in normal mammary epithelium [34]. PPARγ expression is reduced in human benign breast disease and cancers correlating with increased cyclin D1 abundance [35]. Breast cancer control animals show low level of PPARγ expression (Fig. 4 lane a), where as Rosiglitazone treated animals show increased PPARγ expression (Fig. 4 lane b). However, cisplatin treated (Fig. 4 lane c) rats showed low level of PPARγ expression. Pretreatment of rosiglitazone show maximum increase in expression of PPARγ (Fig. 4 lane d) which very well coincides with maximum antitumour activity.

### Change in histone post-translational modifications by pretreatment of rosiglitazone in breast cancer rats

**Figure 5:**
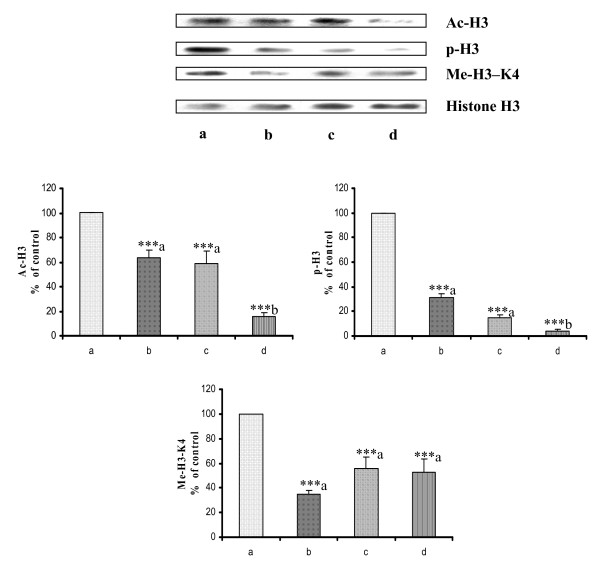
**Western blot of histone modifications in mammary tumours**. Western blot of acetylation, phosphorylation and methylation levels of histone H3 in mammary tumours after treatment with Rosiglitazone and Cisplatin in DMBA induced breast cancer rats. Where, lane a: Cancer Control, lane b: Rosiglitazone, lane c: Cisplatin and lane d: Rosiglitazone pre-treatment. Results were normalized with respect to total histone H3. Similar results were obtained in three independent set of experiments. All the values were represented as Mean ± S.E.M. (n = 3), **P < 0.01; * P < 0.05; a Vs Cancer Control and b Vs Cisplatin.

The modification of the lysine groups of core histones by multiple post-translational events including phosphorylation and acetylation coincident with activation of mitogenic signalling [36]. In the present study we found increased levels of H3 acetylation and phosphorylation in cancer control rats (Fig 5 lane a). In case of rosiglitazone and cisplatin treated rats (Fig 5 lane b and c) there are decreased levels of histone H3 acetylation and phosphorylation. Rosiglitazone pre-treatment showed maximum decrease in H3 acetylation and phosphorylation in breast cancer animals (Fig. 5 lane d). This decrease in histone H3 acetylation and phosphorylation in combination group clearly indicates that there was decreased cell proliferation and increased differentiation which can be well correlated with maximum tumour reduction in combination therapy. Histone H3 methylation can be equally associated with either transcriptional activation or repression. Methylation of the lysine residue Lys4 of histone H3 (H3-K4) correlates with activation of gene expression [20]. Cancer control group show increased Lys4 methylation (Fig 5 lane a) that can be correlated with increased transcription of cancer causing genes. Our combination of rosiglitazone and cisplatin showed decreased Lys4 methylation of histone H3 (Fig. 5 lane d). Suggesting that there may be decrease in over expression of genes involved in cancer. Indicating that combination of rosiglitazone with cisplatin is more efficacious than individual therapy.

## Discussion

In this study, we provide evidence that rosiglitazone significantly increases the level of PPARγ in mammary tumours, leading to significant reduction in tumour volume. Moreover, combination of rosiglitazone with cisplatin led to remarkable reduction in nephropathy. Cytokines, particularly tumour necrosis factor-α (TNF-α), appears to contribute to cisplatin-induced renal injury and coordinate the activation of a large network of chemokines and cytokines in the kidney following cisplatin injection [9]. Agonists of the peroxisome proliferator-activated receptor-γ (PPARγ), such as rosiglitazone, have been recently reported to regulate inflammation by modulating the production of inflammatory mediators and adhesion molecules [17].

Plasma albumin, creatinine and blood urea nitrogen parameters are considered as an index of nephrotoxicity [37]. Our combination of rosiglitazone pretreatment with cisplatin significantly reduced cisplatin induced nephrotoxicity by lowering the levels of nephrotoxicity markers, BUN and creatinine. Histopathological examination also supports that rosiglitazone pre-treatment prevents cisplatin nephrotoxicity which is clearly evident from the reduced glomerular thickening and vacuolation. In line with the protective effect of rosiglitazone on cisplatin induced nephrotoxicity rosiglitazone pre-treatment also showed significant decrease in TNF-α level. TNFα leads to activation of NFκB which further activates phosphorylation and consequent degradation of inhibitory protein IκBα, leading to release of inflammatory mediators [8]. Thus, reduction in TNFα levels confirms the protection observed in nephrotoxicity and supports our biochemical and histopathological data.

PPARγ is a transcription factor belonging to the nuclear receptor superfamily and forms functional heterodimers with the retinoid × receptor [38,39]. Recently, it has been shown that activation of PPARγ by J2 series cyclopentenone prostaglandins (cyPGs), especially 15-deoxy-D^12,14^-prostaglandin J2 (15d-PGJ2) or synthetic agents, such as antidiabetic thiazolidinediones, causes anti-proliferation, apoptosis, differentiation, and anti-inflammation of certain types of cancer cells [40]. In breast tissue, agonists of PPARγ have been shown to inhibit cell growth, reduce oestrogen production by adipose tissue, inhibit oestrogen receptor (ER) activity and play a role in tumour regression [41,42]. In our study, the PPARγ agonist rosiglitazone activated PPARγ and inhibited progression of breast cancer. Moreover, pretreatment of rosiglitazone with cisplatin showed maximum increase in PPARγ expression which was well correlated with maximum percentage of tumour inhibition.

Deregulated growth signaling pathways and acquired resistance toward apoptosis constitute two hallmarks of most, if not all, human tumours [43]. Activity of MAPkinase p38 is regulated through Akt and is deregulated partly due to Akt activation in human cancer. Activation of Akt antagonizes p38 activation while inactivation of Akt results in p38 activation [33]. Mice deficient in p38α are prone to cancer development using carcinogen or oncogene-induced cancer models. p38α can suppress cell proliferation by antagonizing the JNK/c-Jun pathway, which is an important regulator of proliferation and apoptosis [32]. Our data indicates a significant decrease in expression of p38 in breast cancer control animals. However, increase in p38 expression by pretreatment of rosiglitazone, suggests an increase in apoptosis of breast cancer cells.

Post-translational modification of histones alters chromatin structure, facilitating the binding of nuclear factors that mediate DNA repair, transcription, and other processes. The modification of the lysine groups of core histones by multiple post-translational events including phosphorylation and acetylation coincident with activation of mitogenic signalling [36]. Increase in acetylation and phosphorylation of histone H3 in cancer cells shows increased cell proliferation and decreased cell differentiation. Breast cancer control animals showed increased phosphorylation and acetylation of histone H3. But rosiglitazone pre-treatment prevented an increase in acetylation and phosphorylation of histone H3. This is first report which shows that rosiglitazone directly or indirectly can induce change in histone H3 modification. Phosphorylation of histone H3 is known to be involved in cell proliferation. Decrease in histone H3 phosphorylation and acetylation by cisplatin and rosiglitazone pretreatment therapy supports our earlier data of maximum tumour volume reduction in the combination therapy. Lys4 methylation of histone H3 is found to be increased in cancer cell which leads to transcription of various oncogenes, our data also showed high level of Lys4 methylation of histone H3 in cancer control animals. However, pretreatment of rosiglitazone with cisplatin showed decreased level of lys4 methylation of histone H3 which suggests that there is decrease in transcription of proto-oncogenes in mammary tumours.

## Conclusion

Combination of rosiglitazone pretreatment with cisplatin leads to reduced nephrotoxicity with a significant increase in antineoplastic activity. Thus this combination can lead to development of new therapeutic regimen with low nephrotoxicity and high antitumour activity against breast cancer. Despite the preponderance of both drugs as sole agents for anti-neoplastic effect, the combinatorial aid of both drugs seems to abate the tumour volume by reducing cell proliferation to an appreciable extent.

## Abbreviations

DMBA: 7,12-dimethyl benz{a}anthracene; NFκB: Nuclear factor κB; TNFα: Tumour necrosis factor α; BUN: Blood Urea Nitrogen; PPARγ: Peroxisome proliferator-activator receptor.

## Competing interests

The authors declare that they have no competing interests.

## Authors' contributions

KT planned and supervised the project and has given final approval of the version to be published. PCB planned and performed all the *in vivo *experiments and has made substantial contributions to analysis and interpretation of data. JG has carried out immunoblotting experiments and its analysis and also involved in drafting the manuscript.

## Pre-publication history

The pre-publication history for this paper can be accessed here:

http://www.biomedcentral.com/1471-2407/9/107/prepub
